# The C-Terminal V5 Domain of Protein Kinase Cα Is Intrinsically Disordered, with Propensity to Associate with a Membrane Mimetic

**DOI:** 10.1371/journal.pone.0065699

**Published:** 2013-06-06

**Authors:** Yuan Yang, Tatyana I. Igumenova

**Affiliations:** Department of Biochemistry and Biophysics, Texas A & M University, College Station, Texas, United States of America; University of South Florida College of Medicine, United States of America

## Abstract

The C-terminal V5 domain is one of the most variable domains in Protein Kinase C isoforms (PKCs). V5 confers isoform specificity on its parent enzyme through interactions with isoform-specific adaptor proteins and possibly through specific intra-molecular interactions with other PKC domains. The structural information about V5 domains in solution is sparse. The objective of this work was to determine the conformational preferences of the V5 domain from the α isoform of PKC (V5α) and evaluate its ability to associate with membrane mimetics. We show that V5α and its phosphorylation-mimicking variant, dmV5α, are intrinsically disordered protein domains. Phosphorylation-mimicking mutations do not alter the overall conformation of the polypeptide backbone, as evidenced by the local nature of chemical shift perturbations and the secondary structure propensity scores. However, the population of the “cis-trans” conformer of the Thr^638^-Pro^639^-Pro^640^ turn motif, which has been implicated in the down-regulation of PKCα via peptidyl-prolyl isomerase Pin1, increases in dmV5α, along with the conformational flexibility of the region between the turn and hydrophobic motifs. Both wild type and dmV5α associate with micelles made of a zwitterionic detergent, n-dodecylphosphocholine. Upon micelle binding, V5α acquires a higher propensity to form helical structures at the conserved “NFD” motif and the entire C-terminal third of the domain. The ability of V5α to partition into the hydrophobic micellar environment suggests that it may serve as a membrane anchor during the PKC maturation process.

## Introduction

**Figure 1:**
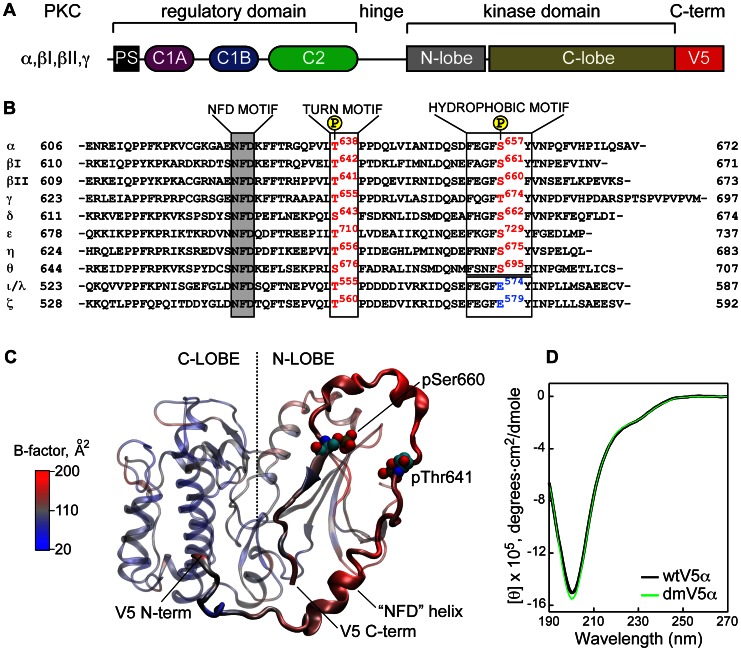
V5 domain is a variable C-terminal region of PKCs. (**A**) Linear diagram of conventional PKC isoforms illustrating the multi-modular structure of the enzyme. (**B**) Alignment of V5 primary structures of PKC isoforms from *M. musculus*. The conserved NFD motif is highlighted in gray; HM and TM motifs are boxed. The Ser/Thr residue of the HM is a Glu residue (blue) in atypical isoforms. (**C**) Catalytic domain (residues 339–679) taken from the crystal structure of the PKCβII “intermediate”, PDB ID 3PFQ. The B-factors of backbone atoms are mapped onto the structure as a color gradient. The N−/C-lobes of the kinase domain and V5 are shown with transparent and opaque representations, respectively. (**D**) Circular dichroism spectra of micelle-free wtV5α and dmV5α domains.

Protein Kinase C isoenzymes (PKCs) are serine/threonine kinases that play key roles in a multitude of signal transduction pathways [1], [2]. Ten mammalian PKCs are divided into three classes: conventional (α, βI, βII, and γ, **Figure 1(A)**), which are activated by diacylglycerol and Ca^2+^; novel (δ, ε, η, and θ), which are activated by diacylglycerol only; and atypical (ι/λ and ζ), which are activated by neither cofactor. Through the phosphorylation of their target proteins, PKCs regulate cell differentiation, proliferation, apoptosis, and motility. Altered expression levels of PKC isoforms are implicated in a number of human pathologies, such as cancer [3], cardiac disease [4], diabetes [5], and mood disorders [6]. Modulation of PKC activity in isoform-specific manner – both for therapeutic and research purposes – is one of the current challenges in the PKC field [3], [7].

The catalytic domains of PKCs are highly conserved, with the exception of the most C-terminal region comprising 60–70 amino acids. This region is referred to as the “Variable 5”, or V5, domain. Due to its variability (see **Figure 1(B)**), V5 represents a promising target for the isoform-specific modulation of PKC activity [8]–[12]. Analysis of the existing literature suggests that V5 plays at least three roles within its parent protein: (i) it stabilizes the kinase sub-domain through direct interactions with its N-lobe [13]–[15]; (ii) it participates in the auto-inhibitory interactions with the components of the N-terminal regulatory domain [7], [13], [16]–[18]; and (iii) it mediates sub-cellular localization of PKC isoforms by interacting with the isoform-specific adaptor proteins, receptors for activated C kinases [19].

Newly synthesized (“immature”) PKC partitions into an unidentified membrane cellular compartment, from which it is released in cytosol upon completion of three ordered phosphorylation reactions [20]. This is necessary for the conventional PKC isoforms to reach their full catalytic competence, or maturity [21]. The first phosphorylation, catalyzed by phosphoinositide-dependent kinase, PDK-1, occurs at a conserved Thr residue on the activation loop that is not part of the C-terminal V5 region. However, V5 serves as an interaction site between the newly synthesized PKC and PDK-1 [22]. Two other phosphorylation sites belong to the V5 domain and include the Thr residue of the turn motif (TM) and the Ser residue of the hydrophobic motif (HM), as shown in **Figure 1(B)**. Both of these phosphorylation reactions occur after V5 is released by PDK-1. The identity of the kinase(s) responsible for the V5 phosphorylation is still under debate. Experimental evidence supports the essential role of mTORC2 (mammalian target of rapamycin complex 2) in the phosphorylation of the TM [23], [24] and auto-phosphorylation of the HM [25].

Despite the pivotal role of V5 in the maturation and regulation of the parent enzymes, little is known about its structure in the extended conformation of PKCs that is associated either with immature (i.e. un-phosphorylated) or with activated kinase (fully phosphorylated and membrane-bound via its N-terminal regulatory domain). The existing structural information about V5 domains comes exclusively from the five crystal structures of isolated catalytic domains [14], [15], [26]–[28] and the structure of PKCβII that represents an intermediate in the catalytic pathway [13]. All structures are of mature protein species with a full complement of phosphorylated sites. In some structures, the electron density of the V5 region is either missing or poorly defined [26]–[28]. In others [13]–[15], the V5 domain wraps around the N-terminal lobe of the kinase sub-domain, as shown in **Figure 1(C)**. Even in those structures, the B-factors of the V5 segment are elevated compared to other parts of the protein, indicating that V5 has some degree of either static or dynamic disorder (**Figure S1**).

The objective of this work was to characterize the conformational preferences of the V5 domain from PKCα, a conventional PKC isoform. The V5 domain and its variant used in our studies mimic the C-terminal regions of immature and mature PKCα, respectively. The obtained structural information enabled us to evaluate the effect of negative charges introduced into V5 upon the phosphorylation of TM and HM, and to assess the extent of the V5 conformational changes that are required for the enzyme maturation. Unexpectedly, we found that V5 associates with membrane mimetics and thereby acquires partial helical structure. Our findings suggest that V5 may play an important role in anchoring the newly synthesized immature (and possibly a fully activated mature) PKCα to the membranes.

## Materials and Methods

### Preparation of V5α Samples

V5α (residues 606–672 of Protein Kinase Cα, *M. musculus*) was cloned into a pET31b(+) vector (Novagen) as a C-terminal fusion with ketosteroid isomerase (KSI). QuickChange™ protocol (Agilent Technologies) was used to (i) delete three extra nucleotides from each AlwNI restriction site; and (ii) introduce the phosphorylation-mimicking T638E/S657E mutations into the newly constructed wtV5α plasmid. To prepare uniformly [^15^N, ^13^C] or [^15^N]-enriched proteins, we used the re-suspension method of Marley et al. [29] with M9 minimal media containing 3 g/L of [^13^C-6]-D-glucose (or natural abundance glucose) and 1 g/L of ^15^NH_4_Cl (Cambridge Isotopes). The details of the cloning procedure and purification protocol are given in Section S2 of the File S1.

Protein stock solutions were prepared by dissolving lyophilized V5α in the NMR buffer containing 20 mM MES at pH 6.0, 100 mM KCl, 8% D_2_O, and 0.02% NaN_3_. The protein concentration was determined using the bicinchoninic acid protein assay reagent (Thermo Scientific Pierce) with Bovine Serum Albumin (Sigma-Aldrich) as a standard. NMR samples were prepared by diluting the stock solutions to 250 µM [U-^13^C,^15^N] enriched V5α and [U-^15^N] enriched V5α for assignment and relaxation experiments, respectively.

Natural abundance n-dodecylphosphocholine (DPC) and [U-^2^H_38_, 98%] DPC were purchased from Avanti Polar Lipids and Cambridge Isotopes, respectively. Aliquots of DPC stock solutions in chloroform were dried under a slow stream of N_2_ gas and then under vacuum for 2 hours. The DPC film was re-suspended in NMR buffer by vortexing for 1 min to form a clear micelle solution. The micelle and protein stock solutions were then mixed to produce a final concentration of 316 µM [U-^13^C,^15^N] V5α and 100 mM DPC. Natural abundance DPC and [U-^2^H_38_, 98%] DPC were used to prepare the wtV5α and dmV5α samples, respectively.

### Spectroscopy

The circular dichroism spectra were collected using the Jasco J-815 CD instrument on samples containing either 10 µM V5α or 10 µM V5α/10 mM DPC, both in 10 mM potassium phosphate buffer at pH 7.0.

NMR experiments were performed at magnetic field strengths of 11.7 Tesla or 14.1 Tesla, corresponding to the ^1^H Larmor frequencies of 500 and 600 MHz, respectively. The assignment of cross-peaks to the specific residues was carried out using the following triple-resonance NMR experiments: HNCACB [30], CBCA(CO)NH [30], HNCO [31], HN(CA)CO [32] (micelle-free sample only), and C(CO)NH [33] on the V5α samples uniformly enriched with ^15^N and ^13^C. The NMR resonance assignments are deposited in BioMagResBank under accession numbers: 18927 (wtV5α), 18928 (dmV5α), 18929 (micelle-associated wtV5α), and 18930 (micelle-associated dmV5α). The chemical shift perturbation Δ was calculated according to the following equation [34]:

where Δ*δ_H_*, Δ*δ_N_*, Δ*δ_Cα_*, Δ*δ_Cβ_*, and Δ*δ_CO_* are the chemical shift differences between the ^1^H_N_, ^15^N, ^13^Cα, ^13^Cβ and ^13^CO nuclei. In the conformational analysis of Pro-containing segments of V5α, the populations of cis- and trans- conformers were determined from the peak intensities normalized to the combined intensity of all cross-peaks observed for a given residue.

Longitudinal relaxation rate constants (R_1_), transverse relaxation rate constants (R_2_), and {^1^H}-^15^N Nuclear Overhauser Enhancement (NOE) were measured for all spectrally resolved N-H groups of wtV5α and dmV5α using standard methods [35]. Twelve time points ranging from 0.008 to 0.200 s (R_2_) and 0.020 to 0.700 s (R_1_) were collected, three of which were duplicates. The NOE data were acquired in an interleaved manner, with a 3 s saturation period and a 5 s recycle delay. Cross-peak intensities were used to quantify relaxation, and the uncertainties of these intensities were estimated either from the root-mean-square noise level of the base plane (NOE) or from the duplicate measurements (R_1_ and R_2_). The reduced [36], [37] spectral density mapping [38], [39] approach was used to calculate the values of spectral density at 0, ω_N_, 0.87ω_H_, and 0.92ω_H_ MHz. ω_N_ = 50 MHz and ω_H_ = 500 MHz are the ^15^N and ^1^H Larmor frequencies, respectively.

## Results and Discussion

### Recombinant V5α can be Prepared in Quantities Sufficient for Structural Work

Structural studies using NMR spectroscopy require milligram quantities of highly purified isotopically enriched proteins. Heterologous expression of the full-length 67-residue V5α by itself or with soluble fusion partners in *E. coli* produced a protein that was either un-inducible or severely proteolyzed. Therefore, we directed the expression of V5α into inclusion bodies using the approach pioneered by Walsh’s laboratory [40]. The codon-optimized DNA sequence corresponding to the V5 domain of PKCα (*M. musculus)* was cloned as a fusion with ketosteroid isomerase (KSI) gene into a pET31b(+) expression vector, in which Met codons are inserted between the KSI, the V5 gene, and the (His)_6_ cassette. The fusion protein was extracted from the inclusion bodies, purified using Ni-NTA affinity resin, and cleaved with CNBr. V5 was separated from KSI using dialysis against an aqueous buffer solution and subsequently purified using anion-exchange chromatography. We obtained ∼6–12 mg of >95% pure protein per 1 Liter of cell culture, depending on the expression medium. The detailed expression and purification protocol is given in Materials and Methods.

We prepared two variants of V5: the wild type (wtV5α) and the double mutant mimicking the fully phosphorylated state (dmV5α). The C619S mutation was introduced into both constructs to prevent carbamylation of the Cys residue under the acidic conditions of the CNBr cleavage reaction. The dmV5α contains two mutations: T638E and S657E, where the Glu residue at each position mimics the phosphorylated states of TM and HM (see **Figure 1(B)**). These or equivalent phosphorylation-mimicking mutations were shown to preserve the catalytic competency of both PKCα [41], [42] and PKCβII [43], [44].

### V5α is Intrinsically Disordered with a Propensity to Form α-helical and β Structures

We first used circular dichroism (CD) spectroscopy to evaluate the conformational preferences of the V5α variants. The spectra shown in **Figure 1(D)** are essentially identical for the wtV5α and dmV5α, indicating that the introduction of negative charges at the TM and HM does not appreciably influence the secondary structure content of the domain. The spectra have a pronounced minimum at 200 nm that is typical for intrinsically disordered proteins [45], [46]. There is also a small shoulder at 230 nm suggesting that V5α may have some secondary structure content. Estimation of the secondary structure by the CONTIN [47] software package revealed 4% and 9% content of the regular α-helical and β-structures, respectively.

**Figure 2:**
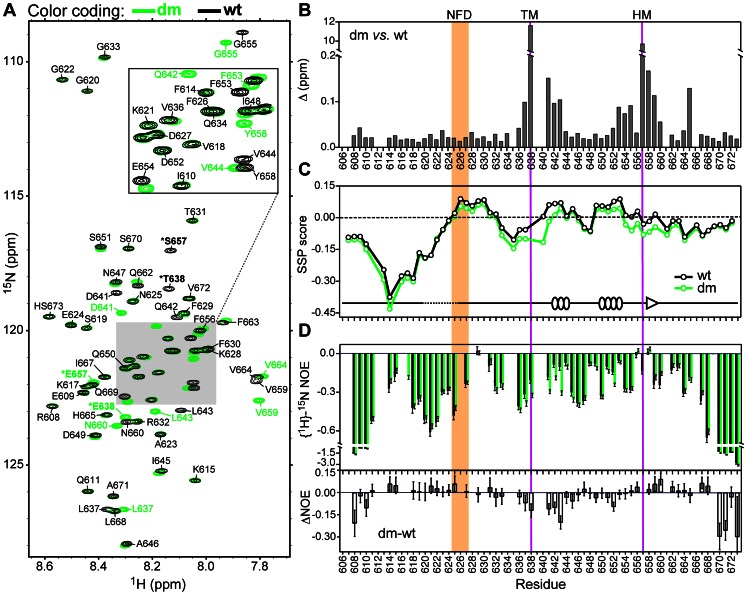
Conformational preferences and sub-nanosecond dynamics of wtV5α and dmV5α. (**A**) Overlay of the ^15^N-^1^H HSQC spectra of wtV5α (black) and dmV5α (green). The asterisks indicate the mutation sites, Thr638 (TM) and Ser657 (HM). HS673 stands for homoserine lactone, which is the C-terminal residue generated upon CNBr cleavage of the (His)_6_-tag from V5α. (**B**) Chemical shift perturbation analysis of the dmV5α-wtV5α pair. Purple vertical lines mark the mutation sites; the NFD motif is shaded. (**C**) SSP scores plotted as a function of the primary structure. The secondary structure elements of the V5 domain in the structure of the catalytic domain from PKCα (PDB ID 3IW4) are shown for comparison. (**D**) Comparison of the hetero-nuclear {^1^H}-^15^N NOE values obtained for wtV5α (black) and dmV5α (green). The NOE values and their difference are plotted against the V5 primary structure in the top and bottom panels, respectively.

The ^15^N-^1^H hetero-nuclear single-quantum coherence (HSQC) spectra of the uniformly [^15^N-enriched] V5α variants are superimposed in **Figure 2(A)**. The cross-peaks in the spectra correspond to the amide ^15^N-^1^H groups of the protein backbone. The assignment of cross-peaks to the specific residues was carried out using the triple-resonance NMR experiments on the V5α samples uniformly enriched with ^15^N and ^13^C. The HSQC spectra of **Figure 2(A)** are characterized by small chemical shift dispersion in the amide ^1^H region, which is a spectroscopic signature of intrinsically disordered proteins [48]. To evaluate the influence of phosphorylation-mimicking mutations, we carried out a chemical shift perturbation (CSP) analysis for the dmV5α-wtV5α pair using the ^1^H_N_, ^15^N, ^13^CO, ^13^Cα, and ^13^Cβ chemical shifts. Significant perturbations are observed at the mutation sites, Ser657 and Thr638, due to the change in amino acid identities. In addition, the residues adjacent to the phosphorylation-mimicking mutations experienced significant chemical shift changes. We attribute those to the changes in the local electrostatic environment caused by the introduction of two negative charges at the TM and HM.

To evaluate the conformational preferences of V5 variants, we calculated secondary structure propensity (SSP) scores [49] using the Cα and Cβ chemical shifts (**Figure 2(C)**). The sign and magnitude of SSP scores, which range from −1 to +1, reflect the propensity of a given residue in the polypeptide to form β- or α-structures. The SSP scores vary significantly across the V5α domain. The N-terminal quarter of both V5α variants has a rather high propensity to form β-structures, with a minimum SSP score reaching −0.43. A region with weak α-helical propensity that contains a conserved NFD motif follows the β segment. In three crystal structures of PKC catalytic domains, the NFD motif forms part of the 8–12 residue helical region. The interaction partners of the NFD motif in the crystalline state vary depending on the PKC isoform/construct and include the adenine ring of ATP (PKCι-ATP complex, 3A8W [14]), the N-terminal lobe of the kinase (catalytic domain of PKCβII, 2I0E [15]), and the regulatory C1B domain (PKCβII intermediate, 3PFQ [13]).

Another V5α segment with weak α-helical propensity is located between the TM and HM, which are marked with purple lines in **Figures 2(B)** **–(D)**. The secondary structure elements of V5α taken from the crystal structure of the catalytic domain of PKCα (PDB ID 3IW4 [28]) are plotted on the SSP graph. Most of V5α is unstructured, and the segment preceding the NFD motif is missing. However, there are two short helical regions between the TM and HM that correlate with positive SSP scores. Taken together, these data suggest that the association of V5α with the N-terminal lobe of the kinase domain during the final step of maturation may include both “conformational selection” and “folding upon binding” mechanisms [50].

To assess the conformational flexibility of the V5α backbone, we measured three relaxation parameters: longitudinal relaxation rate constants (R_1_), transverse relaxation rate constants (R_2_), and {^1^H}-^15^N Nuclear Overhauser Enhancement (NOE) for all spectrally resolved N-H groups (**Figure S2**). As shown in **Figure 2(D)**, the NOE values are negative throughout most of the primary structure. The negative NOE values indicate the dominant contribution of high frequency, i.e. sub-nanosecond, motions to the dynamics of the V5 backbone. The NOE profile of V5 is non-uniform with lower values for the N- and C-termini, and higher values for the short N-terminal segment with high β-structure propensity; the region following the NFD motif; the region upstream of the TM; and the HM. Overall, there is moderate correlation between the regions of lower conformational flexibility, manifested in elevated NOE values, and the regions with a propensity to form secondary structure. The NOE difference graph of **Figure 2(D)** shows that phosphorylation-mimicking mutations increase the conformational flexibility of V5 at the N- and C-termini, as well as between the TM and HM.

To determine the relative contribution of high- and low-frequency motions to the V5 dynamics, we carried out the reduced [36], [37] spectral density mapping [38], [39] analysis of the relaxation data (**Figure S3**). The contributions of high-frequency components are reflected in two spectral density values, J(435 MHz) and J(460 MHz). Both values are rather uniform throughout the V5 backbone but increase towards the N- and C-termini. The contributions of low-frequency motions are reflected in the J(0) and J(50 MHz) terms that show moderate variations across most of the V5 backbone but decrease significantly towards the terminal regions. Overall, the relative average values of spectral densities for both V5 variants are given by J(0):J(ω_N_):J(0.87ω_H_):J(0.92ω_H_) = 17∶9:1∶1. These data are consistent with an extended spectral density frequency profile characteristic of unstructured proteins [51], in which the values of low- and high-frequency components are smaller and larger, respectively, than those of globular proteins of comparable size [36]. The extended spectral density profile, along with the low average values of J(0) (0.60 ns for wtV5α and 0.55 ns for dmV5α), indicate that N-H groups in V5α undergo large-amplitude motions [52].

In summary, high-frequency large-amplitude motions dominate the backbone dynamics of both V5α variants. Regions with weak propensity towards α-helical structure formation, such as the region upstream of the NFD motif and the TM-HM segment, show a higher degree of motional restriction than the rest of the protein residues. Phosphorylation-mimicking mutations increase the conformational flexibility of V5α in the region between the TM and HM, and at the N- and C-termini.

### V5α Samples Multiple Conformations due to Cis–Trans Isomerization of the Peptidyl-prolyl Bonds

**Figure 3:**
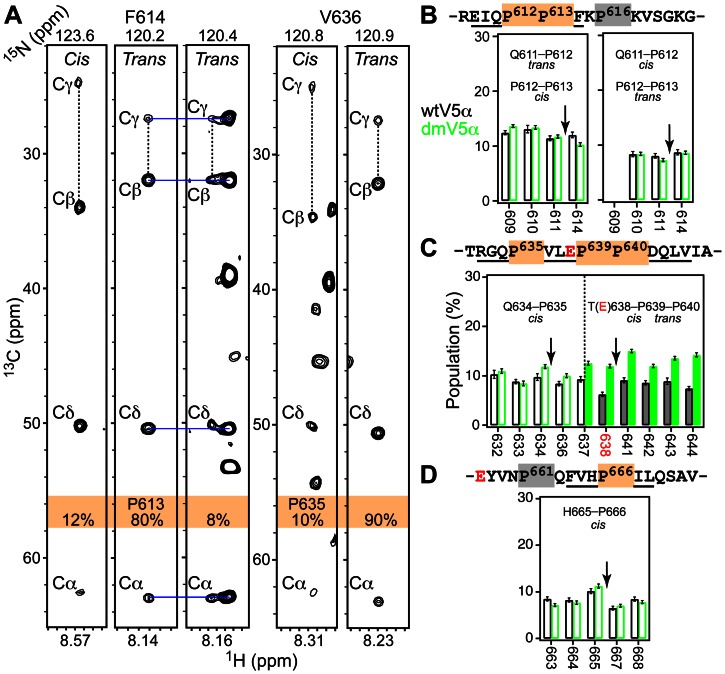
Cis-trans isomerization of Pro residues modulates the conformation of V5α. (**A**) The C(CO)NH strip plots of the F614 and V636 ^1^H-^15^N amide planes showing the characteristic spectroscopic pattern of cis- and trans- Pro^612^-Pro^613^ and Gln^634^-Pro^635^ peptide bonds. The differences between the Cβ and Cγ chemical shifts are marked with vertical dashed lines. Horizontal blue lines indicate the positions of the Cβ and Cγ cross-peaks corresponding to the most abundant trans Pro^612^-Pro^613^ conformer. (**B**)–(**D**) Fractional populations of the V5α species having one Xaa-Pro bond in cis-conformation. Isomerizing Pro residues are highlighted in orange. The wtV5α and dmV5α data are shown in black and green, respectively. The position of Pro residues is indicated with arrows. Residues with quantifiable populations are underlined. In (**C**), the mutation site T638E is highlighted in red. The data for the turn motif Thr(Glu)^638^-Pro^639^-Pro^640^ are shown with filled bars.

The ^15^N-^1^H HSQC spectra of both wtV5α and dmV5α showed a subset of minor cross-peaks. We were able to assign these peaks using three-dimensional NMR experiments. The peaks correspond to the residues that bracket specific prolines in the primary structure of V5α. Given that our V5α preparations are homogeneous, we concluded that both wtV5α and dmV5α sample multiple conformations due to the cis-trans isomerization of Xaa-Pro bonds, where Xaa is the preceding amino acid. These conformations are in slow exchange on the NMR chemical shift timescale. A distinct spectroscopic signature of trans and cis- Xaa-Pro bonds is the difference between Pro Cβ and Cγ chemical shifts, Δ(Cβ-Cγ): 4.5±1.2 ppm and 9.6±1.7 ppm, respectively [53]. This is most conveniently detected in the ^15^N strips of the three-dimensional C(CO)NH spectra that correlate the chemical shifts of the N-H amide group of the residue following Pro to the ^13^C resonances of the Pro sidechain. Representative data are shown in **Figure 3(A)** for the cis- and trans-conformations of P613 and P635.

V5α has a total of eight Pro residues highlighted in **Figures 3(B–D)** **.** We determined that six of them are either involved in (P612, P613, P635, P639, and P666) or influenced by (P640) the cis-trans isomerization process. For the two isolated Pro residues, P635 and P666, the most abundant conformers with a population of ≳ 90% correspond to the trans Gln^634^-Pro^635^ and His^665^-Pro^666^ peptide bonds. The average populations of cis-conformers are 9 (10)% and 8 (8)%, respectively, for the wtV5α (dmV5α). The data for individual residues are given in **Figures 3(C)** and **3(D)**.

The other four prolines occur as pairs, Pro^612^-Pro^613^ and Pro^639^-Pro^640^. Each pair could potentially give rise to four possible conformations due to cis-trans isomerization. We identified a total of three sets of cross-peaks for the residues that bracket the Pro^612^-Pro^613^ segment. Based on the Δ(Cβ-Cγ) values of P613, they correspond to the trans-, cis-, and trans- conformations of the Pro^612^-Pro^613^ peptide bond, with the populations of the latter two given in **Figure 3(B)**. The isomerization state of Gln^611^-Pro^612^ cannot be determined directly because Pro613 does not have an N-H group. However, extensive thermodynamic data on unstructured peptides [54] suggest that the “cis-cis” conformation of Gln^611^-Pro^612^-Pro^613^ would be the least populated of the four. Therefore, we assigned the three V5α species to “trans-trans” (80%), “trans-cis” (12%), and “cis-trans” (8%) conformers of the Gln^611^-Pro^612^-Pro^613^ segment; the populations are comparable in the wtV5α and dmV5α.

In contrast to the Pro^612^-Pro^613^ pair, the conformational exchange behavior of the Pro^639^-Pro^640^ segment that immediately follows the turn motif, T638, differs between wtV5α and dmV5α. In both V5α constructs, we identified two conformers: major and minor. Based on the Δ(Cβ-Cγ) values of P640, both conformers have a trans Pro^639^-Pro^640^ peptide bond. Using the same thermodynamic considerations as above, we assigned the major conformer to the “trans-trans” and the minor conformer to the “cis-trans” conformations of the Thr^638^(Glu)-Pro^639^-Pro^640^ segment. The average population of the minor species increases from 8% in the wtV5α to 13% in the dmV5α (**Figure 3(C)** **, right**).

The shift of the equilibrium towards the cis- peptidyl-prolyl bond in the phosphorylation-mimicking variant is likely a property of the local amino acid context of V5 rather than a general consequence of introducing a negative charge. Indeed, studies of model pentapeptides revealed that having Xaa = Glu instead of Thr does not alter the population of the cis- Xaa-Pro bond [54]. In another peptide study, phosphorylation of the Thr residue slowed down the rate of cis-trans isomerization compared to that of the Thr-Pro, but did not produce a systematic change in the population of the cis-isomers [55]. The conformational preferences of the Thr^638^-Pro^639^ bond may play a role in the Pin1-mediated down-regulation of PKCα. Pin1 is a peptidyl-prolyl isomerase that, according to the recently proposed model [56], docks onto the hydrophobic motif of V5 and catalyzes the cis-trans isomerization of turn motif, pThr^638^-Pro^639^. In this model, the trans-conformer of the pThr638-Pro639 peptidyl-prolyl bond in the agonist-activated PKC is more susceptible to dephosphorylation and ubiquitin-mediated degradation than the cis-conformer. Our NMR data provide direct evidence of the existing equilibrium between the cis- and trans- conformers of the turn motif in V5α. Structural characterization of the Pin1-V5α complex is required to determine the molecular basis of V5α-Pin1 interaction and its dependence on the conformation of the pThr^638^-Pro^639^ bond.

### V5α Binds to DPC Micelles and Acquires Partial α-helical Structure

**Figure 4:**
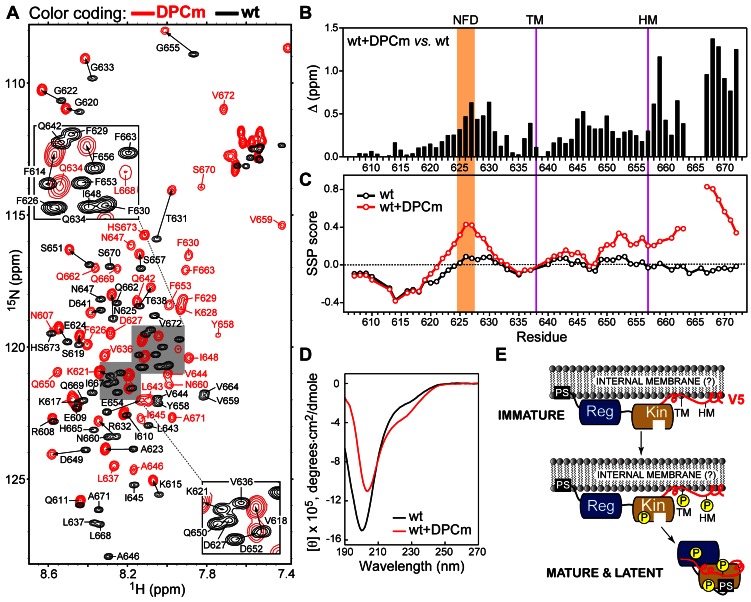
wtV5α associates with DPC micelles (DPCm) and acquires partial α-helical structure. (**A**) Overlay of the ^15^N-^1^H HSQC spectra of wtV5α (black) and wtV5α/DPCm (red). HS673 stands for homoserine lactone, which is the C-terminal residue generated upon CNBr cleavage of the (His)_6_-tag from V5α. (**B**) Chemical shift perturbation analysis of the wtV5α/DPCm and wtV5α pair. Residues having an incomplete set of chemical shifts are listed in Section S4 of the File S1. (**C**) SSP scores plotted as a function of the primary structure. (**D**) Circular dichroism spectra of wtV5α domains in the presence (red) and absence (black) of DPC micelles. (**E**) Proposed model of PKCα maturation, in which V5 serves as a membrane anchor. Reg and Kin are the regulatory and kinase domains, respectively. The regulatory domain comprises the tandem C1A and C1B domains, commonly referred to as C1, and the C2 domain. PS stands for the pseudo-substrate region. V5α is shown in red.

To determine the propensity of V5α to partition into membrane-mimicking hydrophobic environment, we conducted NMR experiments on both variants in the presence of dodecylphosphocholine (DPC) micelles. DPC micelles were chosen as a membrane-mimicking medium because it worked well for the two peripheral membrane domains of PKCα, C1B [57] and C2 (K. A. Morales and T.I. Igumenova, unpublished data). Unexpectedly, the NMR spectra of wtV5α changed dramatically upon addition of DPC micelles (**Figure 4(A)**), resulting in significant cross-peak shifts for all but the most N-terminal amino acids. The changes in cross-peak positions are accompanied by changes in the peak line-widths, indicating that wtV5α binds to the micelles. The same pattern of spectral changes was observed for dmV5α, as shown in **Figure S4(A)**. The backbone resonances of wtV5α and dmV5α complexed to DPC micelles were assigned using triple-resonance NMR experiments and subjected to CSP analysis using the DPC-free shifts as a reference. The residue-specific Δ values of wtV5α (**Figure 4(B)**) are small for the first 15 amino acids, up to the start of the NFD motif at position 625. The chemical shifts of the NFD-containing region (residues 625–634) are significantly perturbed, as well as the region between the TM and HM. Downstream of HM, Δ values increase further reaching the largest values at the C-terminus. Two residues, 664 and 665, are broadened beyond detection indicating a presence of a chemical exchange process that is intermediate on the NMR chemical shift timescale.

SSP values of the micelle-associated wtV5α, calculated based on Cα and Cβ chemical shifts, are plotted against the primary structure in **Figure 4(C)**. The micelle-free wtV5α data are also shown for comparison. According to the SSP data analysis, V5α acquires high propensity to form helical structures in two regions upon binding to DPC micelles. The first region is the NFD motif, where the SSP score reaches a maximum value of 0.43. The second region is the last one-third of the V5α domain starting from residue 649. The helical propensity increases along the polypeptide backbone reaching the maximum of 0.83 at residue 667, and then decreases to 0.34 at the C-terminus. Our findings are further corroborated by the circular dichroism data that we collected for both V5α variants in the presence of 10 mM DPC. The CD spectra of micelle-bound wtV5α are overlaid with the DPC-free spectra in **Figure 4(D)**. The shoulder region between 220 and 230 nm becomes more prominent, indicating an increase in the helical structure content. Estimation by the CONTIN [47] software package produces a ∼two-fold increase in the regular α-helical structure content, from 4 to 9%, compared to the micelle-free wtV5α. The β-structure content remains unchanged. Micelle-associated dmV5α shows a similar pattern of chemical shift perturbations, SSP scores, and CD spectra (see ****Figures S4, (B)–(D)****).

These findings led us to hypothesize about a novel role of the V5 domain in the PKCα maturation process. Immature, or newly synthesized, PKCα is not phosphorylated. It has been shown that immature PKC partitions into the detergent-soluble membrane fraction of the cell [20]; the nature of the membrane compartment (internal membranes or plasma membrane) is not known. The current thinking in the field is that immature PKC is tethered to the membranes via the pseudo-substrate region [58] and weak cofactor-free interactions of the C1 and C2 domains [21]. Together, these domains make up the entire N-terminal half of the enzyme, as shown in **Figure 1A**. The propensity of wtV5α to partition into the hydrophobic membrane-mimicking environment suggests that it can serve as a membrane anchor in immature PKCα, as illustrated in our model (**Figure 4(E)**). In this model, not only the N-terminal regulatory domain and the pseudo-substrate regions are tethered to the membrane, but also the V5 domain, which provides the anchoring point at the C terminus of the enzyme. This additional interaction may stabilize the immature PKCα, increase its lifetime at the membrane, and thereby facilitate the maturation process.

The maturation process involves three ordered phosphorylation reactions, with two phosphorylation sites residing on the conserved hydrophobic and turn motifs, respectively [21]. It is well established that, upon maturation, PKCα dissociates from the membrane into the cytosol. In the cytosol, it adopts a compact latent form, in which the pseudo-substrate region is involved in the auto-inhibitory interactions with the active site of the kinase, as shown in **Figure 4E** [59].

What could be the driving force for the mature (i.e. phosphorylated) V5α to disengage from the membrane and associate with the N-terminal lobe of the kinase? We speculate that this process is driven by the hydrogen-bonding and electrostatic interactions between the phosphate groups of TM and HM and the residues of the N-terminal lobe of the kinase. These interactions are present in several structures of catalytic domains [13]–[15], [26]. Our data on dmV5α show that the interaction mode of V5α with zwitterionic detergent DPC is not influenced by the introduction of negative charges at the HM and TM (compare **Figures 4** and **S4**). If negatively charged lipids are present in the membrane compartment where PKCα undergoes the maturation process, then the electrostatic repulsion between the phosphate groups of TM and HM and the negatively charged lipid head-groups will also promote the dissociation of the phosphorylated V5α from the membrane. As part of the future studies, we are planning to determine the lipid specificity of the V5α-membrane interactions using large unilamellar vesicles with varying lipid composition and fluorescence-based detection of binding.

Upon activation, the compact latent form of the enzyme releases its pseudo-substrate region from the active site of the kinase domain. This domain rearrangement is driven by the C1 and C2 domains undergoing membrane insertion in response to binding their respective cofactors, diacylglycerol (C1 domains) and Ca^2+^/phosphatidylserine (C2 domain). The ability of the dmV5α to associate with micelles suggests that V5 can potentially function as a membrane anchor for the fully activated mature PKC, in which the entire N-terminal regulatory domain is membrane-bound. Such an interaction will be dependent on the lipid composition of the membrane and the availability of other V5 binding partners, such as receptors for activated C kinases.

## Supporting Information

Figure S1 **The B-factors of Cα atoms extracted from the crystal structures of isolated PKCβII catalytic domain (PDB ID 2I0E) and the PKCβII intermediate (PDB ID 3PFQ).** The residues corresponding to the C-terminal V5 domain (609–669) are shaded. V5 has elevated B-factors in both structures, indicating some degree of either static or dynamic disorder.
(TIF)

Figure S2 **(A)** R_1_ and **(B)** R_2_ relaxation rate constants versus the primary structure of V5α. The NFD motif is shaded. The hydrophobic motif (HM) and turn motif (TM) are marked with purple lines.
(TIF)

Figure S3 **(A)** J(0), **(B)** J(50 MHz), **(C)** J(435 MHz), and **(D)** J(460 MHz) versus the primary structure of V5α. The NFD motif is shaded. The hydrophobic motif (HM) and turn motif (TM) are marked with purple lines.
(TIF)

Figure S4 **dmV5α binds to DPC micelles (DPCm) and acquires partial α-helical structure.** **(A)** Overlay of the ^15^N-^1^H HSQC spectra of dmV5α (green) and dmV5α/DPCm (blue) collected at 11.7 Tesla. The DPC concentration is 100 mM. The cross-peaks are labeled according to the residue identity and number. HS673 stands for homoserine lactone, which is the C-terminal residue generated upon CNBr cleavage of the (His)_6_-tag from V5α. **(B)** Chemical shift perturbation analysis of the dmV5α and dmV5α/DPCm pair. The chemical shift perturbation Δ was calculated based on the ^1^H_N_, ^15^N, ^13^Cα, ^13^Cβ and ^13^CO chemical shifts. Residues having an incomplete set of chemical shifts are listed in Section S4. Purple vertical lines indicate the turn and hydrophobic motifs. The NFD motif is shaded. **(C)** SSP scores plotted as a function of the primary structure. Compared to the micelle-free dmV5α, the helical propensity increases for the NFD motif and the surrounding region, the region between the TM and HM, and the most C-terminal amino acid stretch. **(D)** CD spectra of dmV5α in the presence (blue) and absence (green) of DPC micelles. The data were collected using the Jasco J-815 CD instrument on samples containing 10 µM dmV5α, 10 mM DPC in 10 mM potassium phosphate buffer at pH 7.0.
(TIF)

File S1 **Supporting information file that contains: plots of B-factors of the V5 domain from PKCβII; cloning and purification protocol for the V5α constructs; backbone relaxation parameters and spectral density values of wtV5α and dmV5α; wtV5α/DPC and dmV5α/DPC residues excluded from the CSP analysis; and NMR spectra showing the binding of dmV5α to DPC micelles.**
(PDF)
