# Supplementary figures and images for: The C-Terminal V5 Domain of Protein Kinase Cα Is Intrinsically Disordered, with Propensity to Associate with a Membrane Mimetic
Source: PLoS One. 2013 Jun 6;8(6):e65699. doi: 10.1371/journal.pone.0065699 (PMC3675085; doi:10.1371/journal.pone.0065699)

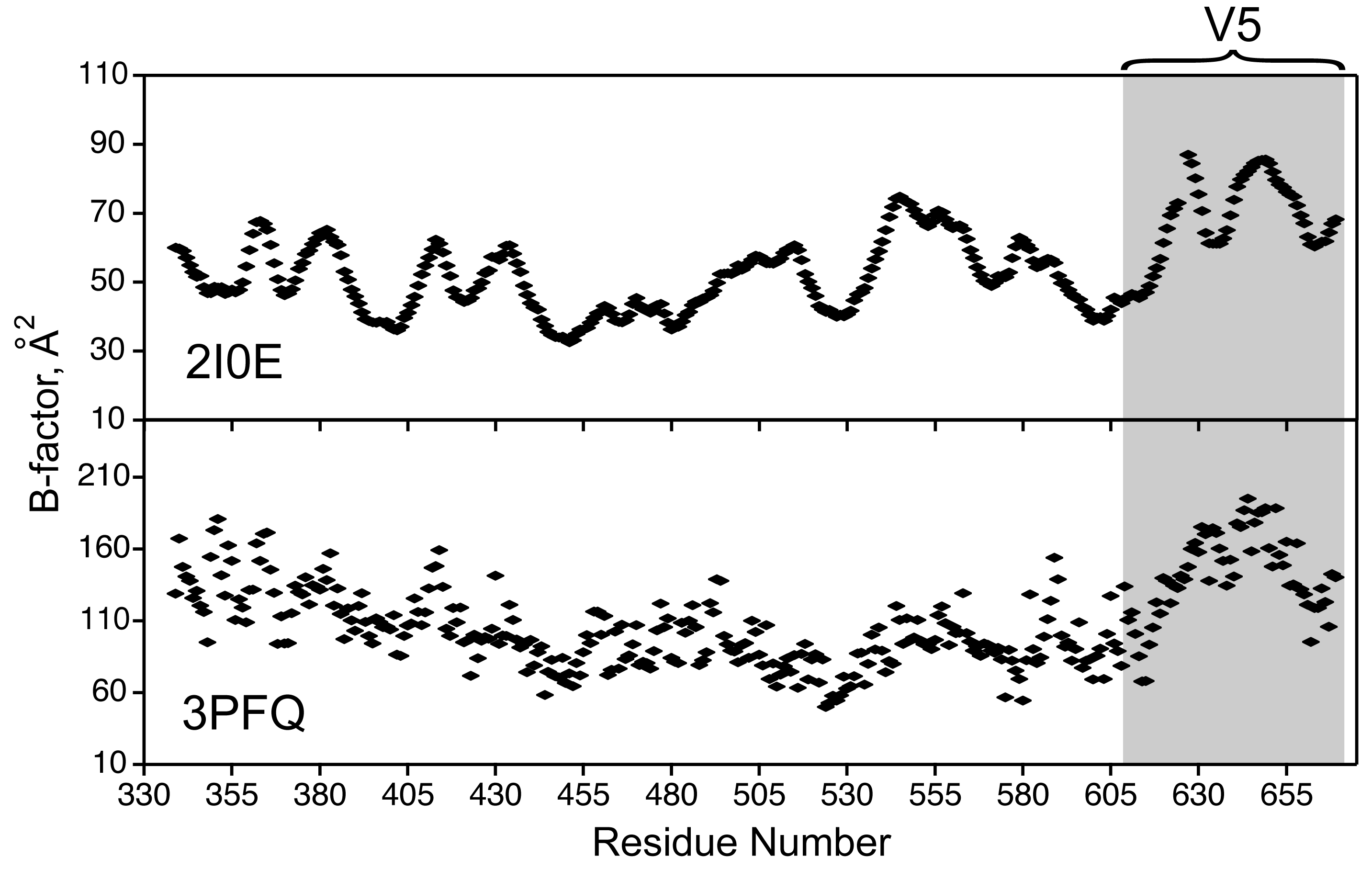

Supplement: Figure S1 — The B-factors of Cα atoms extracted from the crystal structures of isolated PKCβII catalytic domain (PDB ID 2I0E) and the PKCβII intermediate (PDB ID 3PFQ). The residues corresponding to the C-terminal V5 domain (609–669) are shaded. V5 has elevated B-factors in both structures, indicating some degree of either static or dynamic disorder. (TIF) [file pone.0065699.s001.tif]

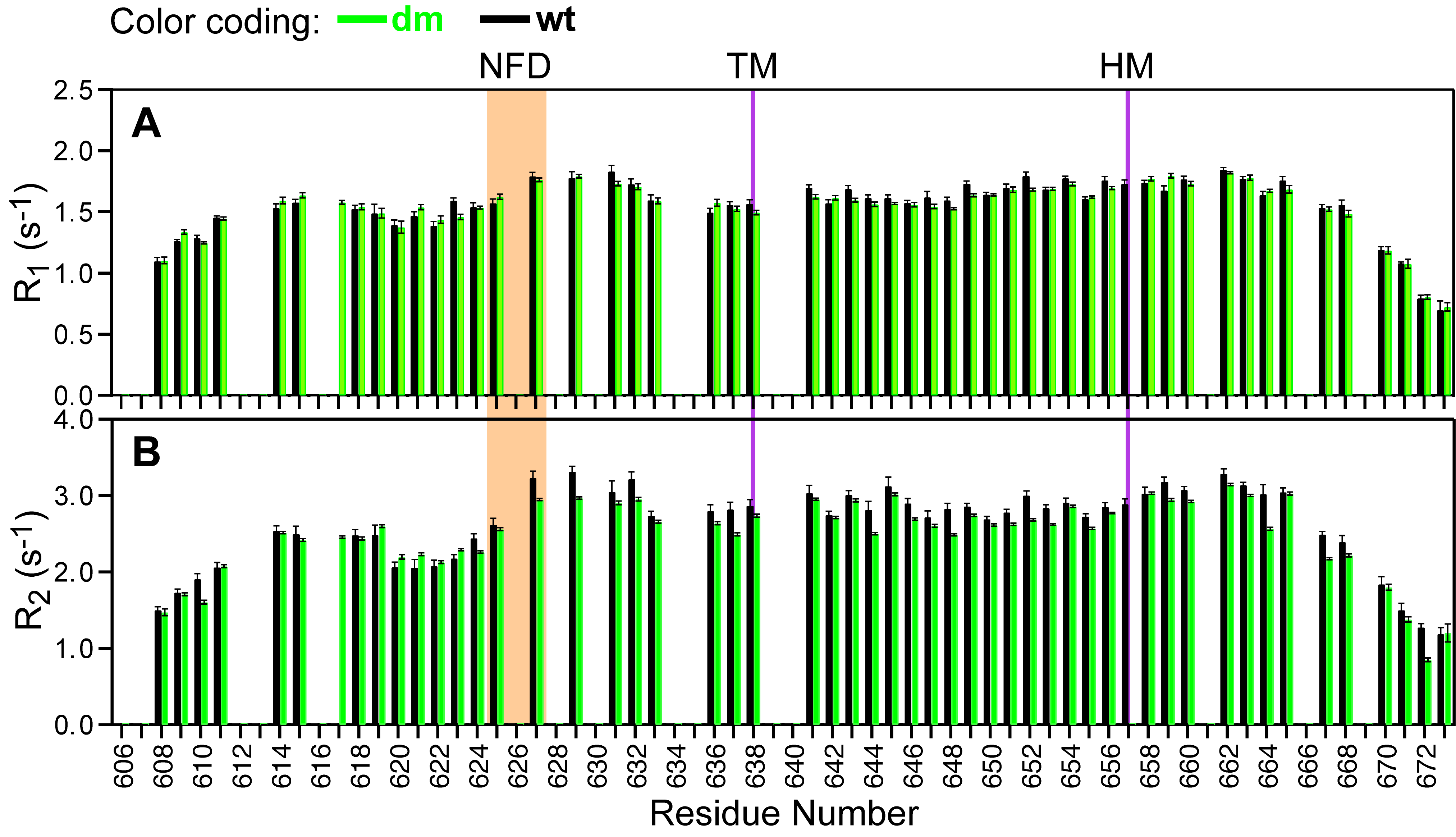

Supplement: Figure S2 — (A) R1 and (B) R2 relaxation rate constants versus the primary structure of V5α. The NFD motif is shaded. The hydrophobic motif (HM) and turn motif (TM) are marked with purple lines. (TIF) [file pone.0065699.s002.tif]

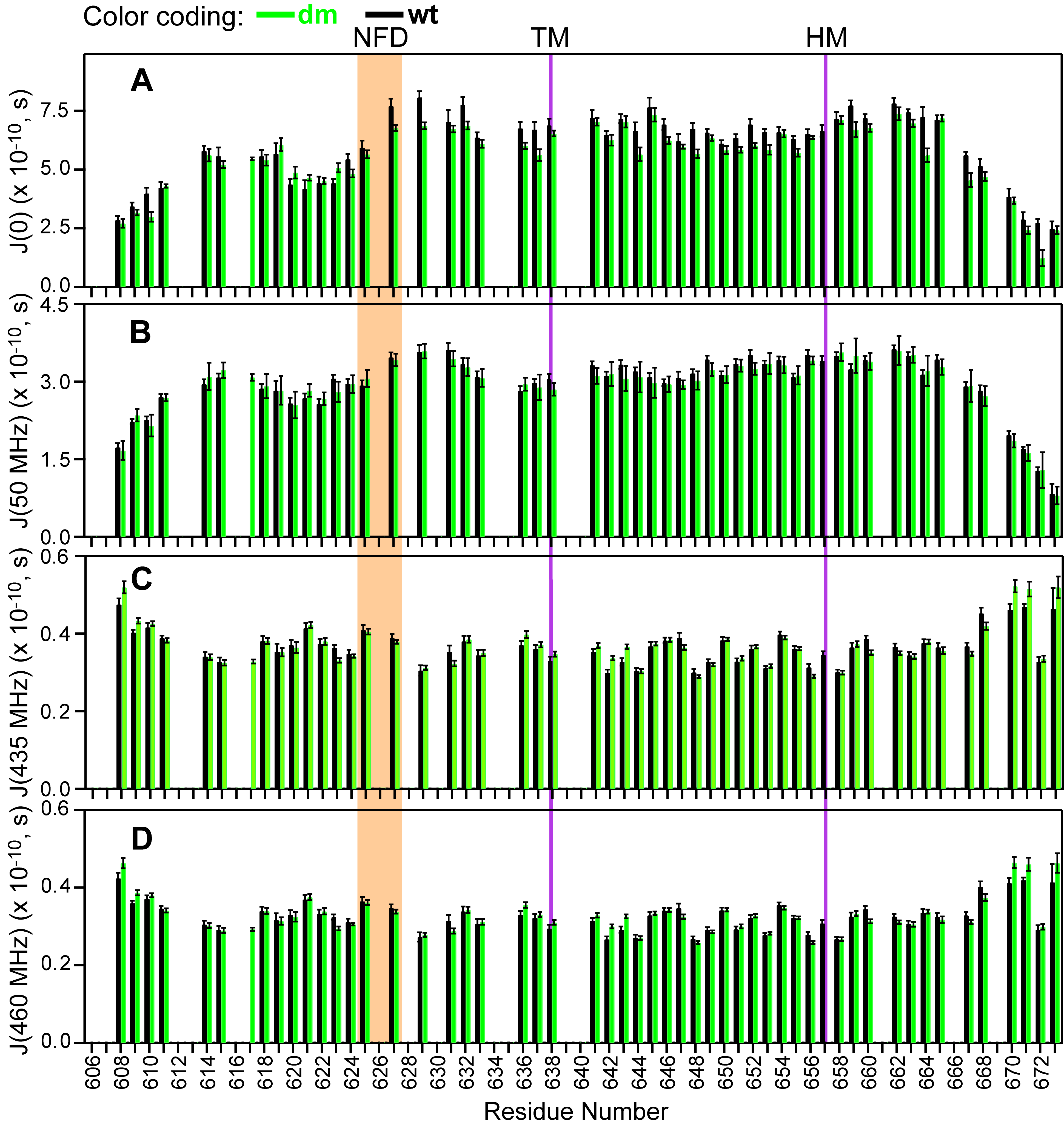

Supplement: Figure S3 — (A) J(0), (B) J(50 MHz), (C) J(435 MHz), and (D) J(460 MHz) versus the primary structure of V5α. The NFD motif is shaded. The hydrophobic motif (HM) and turn motif (TM) are marked with purple lines. (TIF) [file pone.0065699.s003.tif]

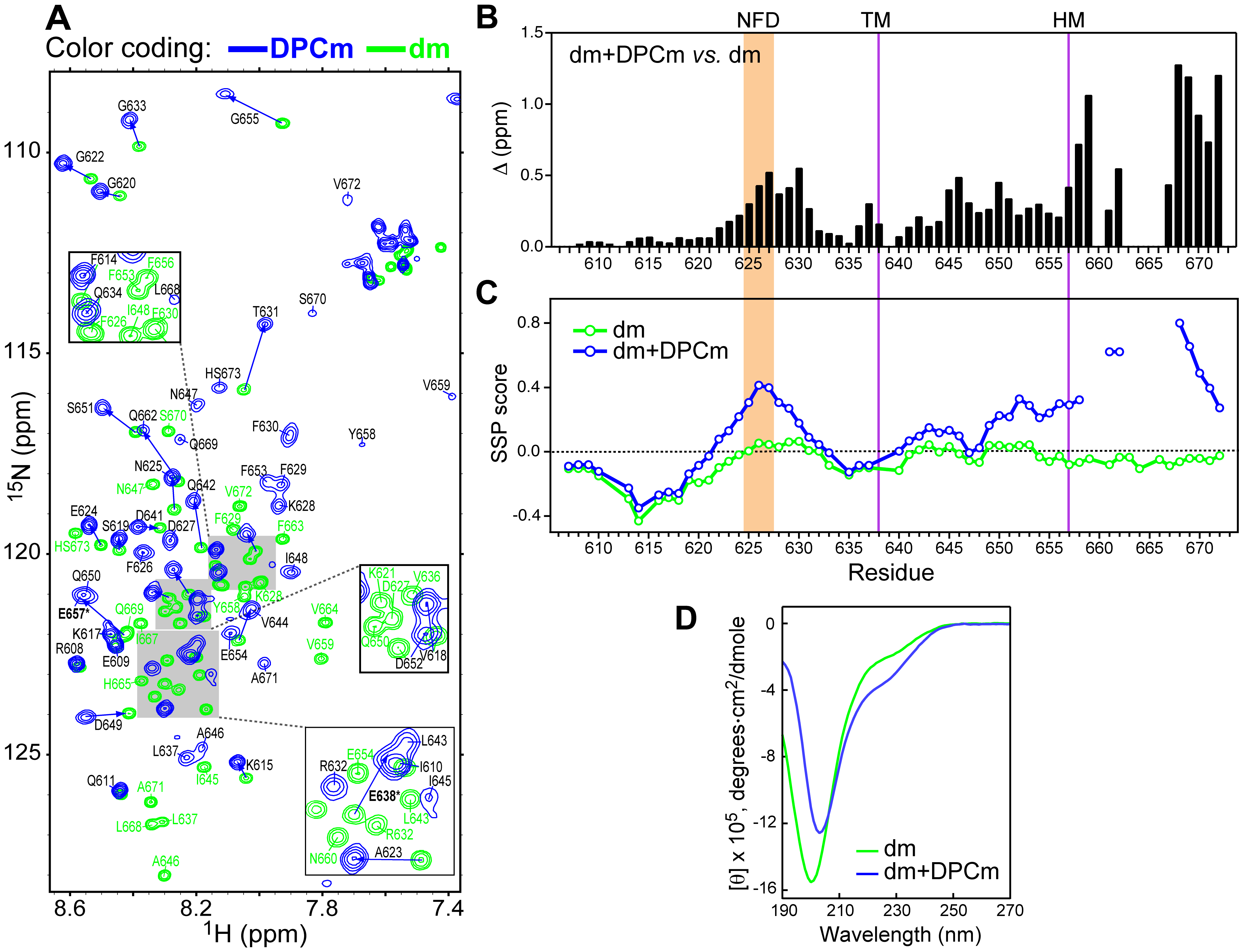

Supplement: Figure S4 — dmV5α binds to DPC micelles (DPCm) and acquires partial α-helical structure. (A) Overlay of the 15N-1H HSQC spectra of dmV5α (green) and dmV5α/DPCm (blue) collected at 11.7 Tesla. The DPC concentration is 100 mM. The cross-peaks are labeled according to the residue identity and number. HS673 stands for homoserine lactone, which is the C-terminal residue generated upon CNBr cleavage of the (His)6-tag from V5α. (B) Chemical shift perturbation analysis of the dmV5α and dmV5α/DPCm pair. The chemical shift perturbation Δ was calculated based on the 1HN, 15N, 13Cα, 13Cβ and 13CO chemical shifts. Residues having an incomplete set of chemical shifts are listed in Section S4. Purple vertical lines indicate the turn and hydrophobic motifs. The NFD motif is shaded. (C) SSP scores plotted as a function of the primary structure. Compared to the micelle-free dmV5α, the helical propensity increases for the NFD motif and the surrounding region, the region between the TM and HM, and the most C-terminal amino acid stretch. (D) CD spectra of dmV5α in the presence (blue) and absence (green) of DPC micelles. The data were collected using the Jasco J-815 CD instrument on samples containing 10 µM dmV5α, 10 mM DPC in 10 mM potassium phosphate buffer at pH 7.0. (TIF) [file pone.0065699.s004.tif]
