# Supplementary material for: The C-Terminal V5 Domain of Protein Kinase Cα Is Intrinsically Disordered, with Propensity to Associate with a Membrane Mimetic
Source: PLoS One. 2013 Jun 6;8(6):e65699. doi: 10.1371/journal.pone.0065699 (PMC3675085; doi:10.1371/journal.pone.0065699)
Supplement: File S1 — Supporting information file that contains: plots of B-factors of the V5 domain from PKCβII; cloning and purification protocol for the V5α constructs; backbone relaxation parameters and spectral density values of wtV5α and dmV5α; wtV5α/DPC and dmV5α/DPC residues excluded from the CSP analysis; and NMR spectra showing the binding of dmV5α to DPC micelles. (PDF) [file pone.0065699.s005.pdf]

SUPPORTING INFORMATION

The C-terminal V5 domain of Protein Kinase Cα is intrinsically disordered, with propensity to associate with a membrane mimetic

Yuan Yang and Tatyana I. Igumenova[†]

Department of Biochemistry and Biophysics, Texas A&M University, 300 Olsen Boulevard, College Station, TX 77843

[†]Corresponding author

E-mail: tigumenova@tamu.edu; Phone: (979) 845 6312; Fax: (979) 845 4946.

S1. B-factors of the V5 domain from PKCβII, a conventional PKC isoform.

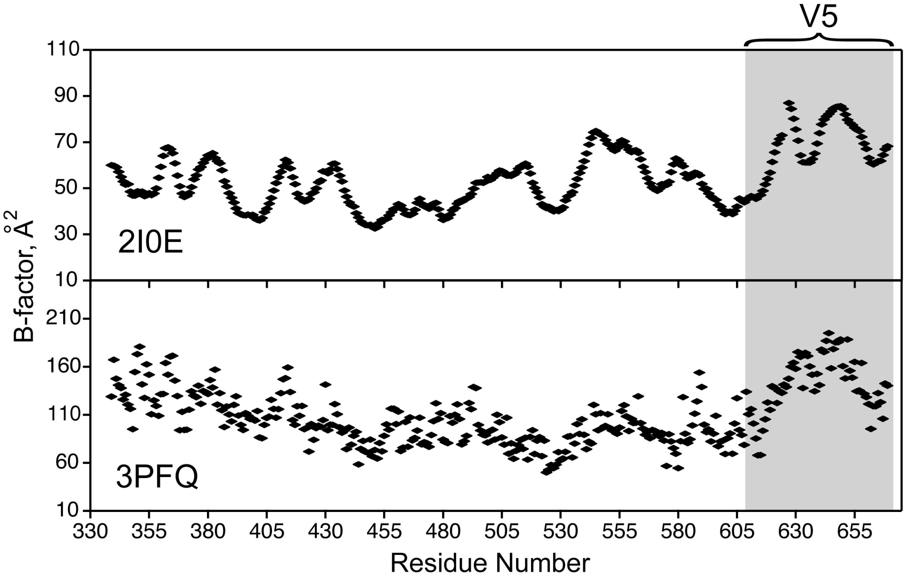

Figure S1. The B-factors of Cα atoms extracted from the crystal structures of isolated PKCβII catalytic domain (2I0E [1]) and the PKCβII intermediate (3PFQ [2]). The residues corresponding to the C-terminal V5 domain (609-669) are shaded. V5 has elevated B-factors in both structures, indicating some degree of either static or dynamic disorder.

S2. Cloning and purification protocol for the V5α constructs.

V5α (residues 606–672 of Protein Kinase Cα, *M. musculus*) was cloned into a pET31b(+) vector (Novagen) as a C-terminal fusion with ketosteroid isomerase (KSI) using the following protocol. A codon-optimized DNA sequence of V5α in a pUC57 vector was obtained from Genscript. Two identical AlwNI restriction sites with Met codons, 5'-CAGATGCTG-V5α-CAGATGCTG-3', were engineered into the sequence. In addition, C619S mutation was introduced into the gene to prevent V5α dimerization via the formation of disulfide bonds. The source plasmid, pUC57, was digested with the AlwNI restriction enzyme (NEB) at 37 °C for 4 hours. The DNA fragment containing V5α was purified from the 2% agarose gel using the Gel Extraction Kit (Qiagen). The target vector, pET31b(+), was linearized by treatment with AlwNI restriction enzyme at 37 °C for 4 hours and dephosphorylated with alkaline phosphatase (NEB). The V5α DNA insert was ligated into the pET31b(+) vector using T4 DNA ligase (NEB). As a

result, the V5α sequence was placed downstream of the 125 amino acids KSI gene and upstream of a His•tag sequence. QuickChange^{TM} protocol (Agilent Technologies) was used to (i) delete three extra nucleotides from each AlwNI restriction site; and (ii) introduce the phosphorylation-mimicking T638E/S657E mutations into the newly constructed wtV5α plasmid. In this manuscript, we refer to the T638E/S657E mutant as dmV5α.

The fusion proteins KSI–(wtV5α or dmV5α)–His•tag, with KSI, V5α, and His•tag separated by Met residues, were expressed in BL21(DE3) pLysS *E. coli* cells. For natural abundance preparations, the expression was induced by adding isopropyl β-D-1-thiogalactopyranoside (IPTG) to a final concentration of 0.5 mM to the cell cultures at OD_{600} of 0.5. The induction period was 5 hours at 37 °C. For uniformly [^{15}N, ^{13}C] or [^{15}N]-enriched proteins, we used the re-suspension method of Marley et al. [3] with M9 minimal media containing 3 g/L of [^{13}C-6]-D-glucose (or natural abundance glucose) and 1 g/L of $^{15}NH_4Cl$ (Cambridge Isotopes). In this case, the protein expression was induced for 4 hours at 37 °C.

The cells were harvested by centrifugation (30 min, 4,000 rpm at 4 °C) and lysed by sonication in a buffer containing 20 mM Tris-HCl, 0.5 M NaCl, and 5 mM imidazole at pH 7.9 (Buffer A). The inclusion bodies containing KSI-V5-His•tag fusion protein were pelleted by centrifugation (15 min at 14,000 rpm, 4°C) and washed once with 1 M urea in Buffer A. The pellet was then solubilized in Buffer B (8 M urea dissolved in Buffer A) and incubated with Ni-Sepharose resin (GE Healthcare) for 1 hour at room temperature. The resin was washed four times with 16 mM imidazole in Buffer B, followed by the elution of the fusion protein by 300 mM imidazole in the same buffer.

The eluted fraction was acidified by adding HCl to a final concentration of 0.5 M. The fusion protein was cleaved at Met residues with CNBr (Acros Organics). CNBr was dissolved in

acetonitrile and added to the acidified fusion protein to obtain a ~100-fold molar excess. The reaction was carried out in the dark under N_2 at room temperature with constant stirring. After 18 hours, the solvent was removed from the reaction mixture using a rotary evaporator at 28 °C. The solid residue containing the target protein was dissolved in deionized water and neutralized with 10 M KOH. The mixture contained V5α, its fusion partner KSI, His•tag, and un-cleaved fusion protein. All species containing a His•tag moiety were removed from solution by an extra incubation step with Ni-Sepharose resin.

To remove urea, the protein solution was dialyzed into a buffer containing 20 mM Tris-HCl and 0.1 mM EDTA at pH 7.0. The precipitated KSI was spun down at 8,000 rpm for 10 min at 4 °C. The supernatant containing V5α was further purified on a HiTrap Q HP anion exchange column (GE Healthcare) using a linear gradient from 0 to 0.4 M NaCl. The purified V5α protein was dialyzed into deionized water, freeze-dried, and stored at –80 °C. Molecular weights of wtV5α and dmV5α were verified by MALDI-TOF mass spectrometry.

S3. Backbone relaxation parameters and spectral density values of wtV5α and dmV5α.

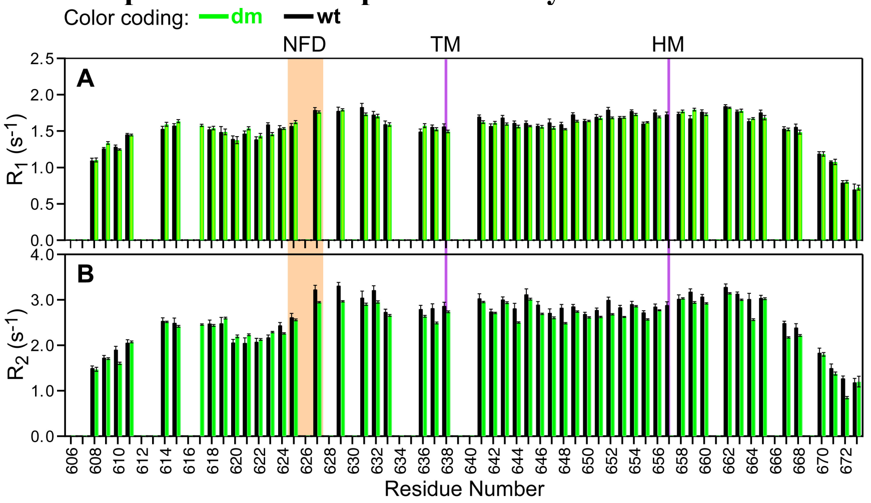

Figure S2. **(A)** R_1 and **(B)** R_2 relaxation rate constants versus the primary structure of V5α. The NFD motif is shaded. The hydrophobic motif (HM) and turn motif (TM) are marked with purple lines.

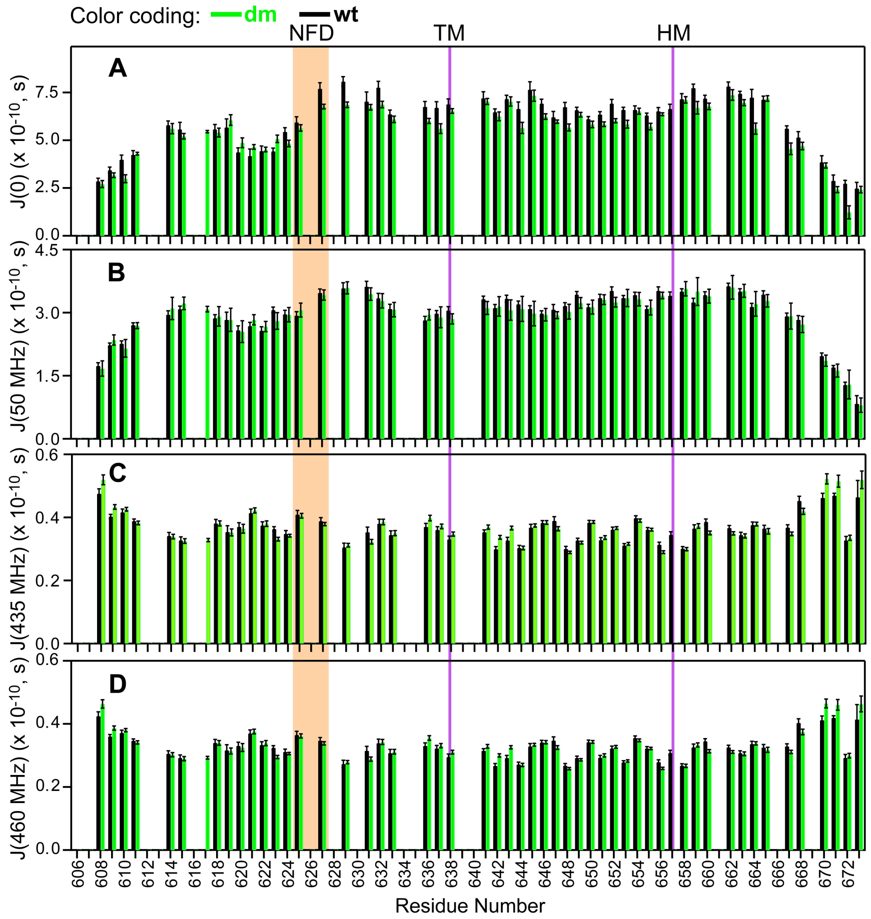

Figure S3. **(A)** J(0), **(B)** J(50 MHz), **(C)** J(435 MHz), and **(D)** J(460 MHz) versus the primary structure of V5α. The NFD motif is shaded. The hydrophobic motif (HM) and turn motif (TM) are marked with purple lines.

S4. wtV5α/DPC and dmV5α/DPC residues that do not have a complete set of shifts for CSP analysis.

The missing/unassigned resonances are indicated in parentheses.

wtV5α: Q611 (^{13}CO), P612 (all), P613 (^{15}N), K615 (^{13}CO), P616 (^{15}N), Q634 (^{13}CO), P635 (^{15}N), T638 (^{13}CO), P639 (all), P640 (^{15}N), N660 (^{13}CO), P661 (^{15}N), F663 (^{13}CO), V664 (all), H665 (all), P666 (all), I667 (^{1}H$_N$, ^{15}N).

dmV5α: Q611 (^{13}CO), P612 (all), P613 (^{15}N), K615 (^{13}CO), P616 (^{15}N), Q634 (^{13}CO), P635 (^{15}N), E638 (^{13}CO), P639 (all), P640 (^{15}N), V659 (^{13}Cα, ^{13}Cβ, ^{13}CO), N660 (all), P661 (^{15}N), Q662 (^{13}Cβ, ^{13}CO), F663 (all), V664 (all), H665 (all), P666 (all), I667 (^{1}H$_N$, ^{15}N, ^{13}Cα, ^{13}Cβ).

S5. dmV5α binds to DPC micelles (DPCm) and acquires partial α-helical structure.

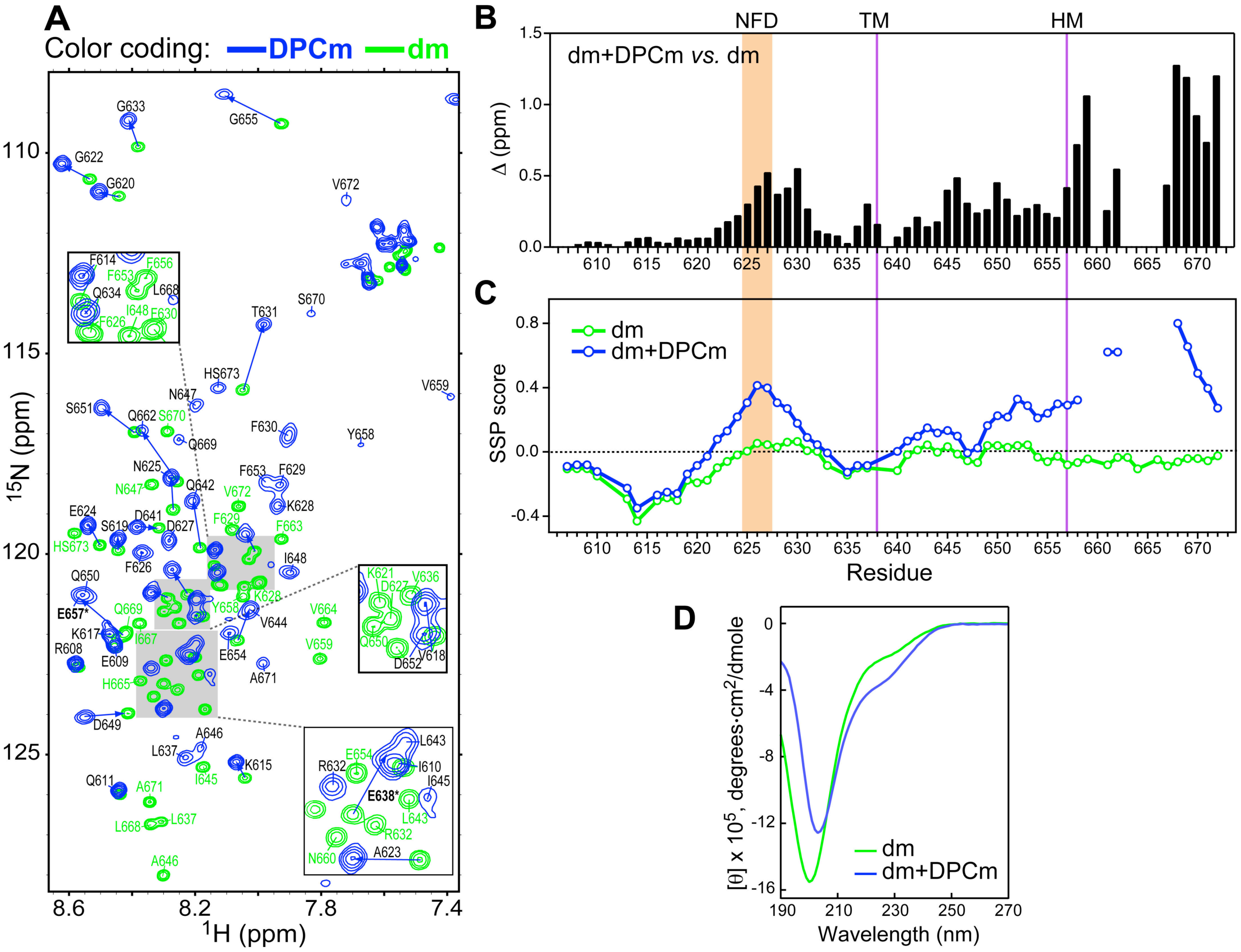

Figure S4. dmV5α binds to DPC micelles (DPCm) and acquires partial α-helical structure. **(A)** Overlay of the ^{15}N-^{1}H HSQC spectra of dmV5α (green) and dmV5α/DPCm (blue) collected at 11.7 Tesla. The DPC concentration is 100 mM. The cross-peaks are labeled according to the residue identity and number. HS673 stands for homoserine lactone, which is the C-terminal residue generated upon CNBr cleavage of the (His)$_6$-tag from V5α. **(B)** Chemical shift perturbation analysis of the dmV5α and dmV5α/DPCm pair. The chemical shift perturbation Δ was calculated based on the $^{1}H_N$, ^{15}N, ^{13}Cα, ^{13}Cβ and ^{13}CO chemical shifts. Residues having an incomplete set of chemical shifts are listed in Section S4. Purple vertical lines indicate the turn and hydrophobic motifs. The NFD motif is shaded. **(C)** SSP scores plotted as a function of the primary structure. Compared to the micelle-free dmV5α, the helical propensity increases for the NFD motif and the surrounding region, the region between the TM and HM, and the most C-terminal amino acid stretch. **(D)** CD spectra of dmV5α in the presence (blue) and absence (green) of DPC micelles. The data were collected using the Jasco J-815 CD instrument on samples containing 10 μM dmV5α, 10 mM DPC in 10 mM potassium phosphate buffer at pH 7.0.

REFERENCES

1. Grodsky N, Li Y, Bouzida D, Love R, Jensen J, et al. (2006) Structure of the catalytic domain of human protein kinase C beta II complexed with a bisindolylmaleimide inhibitor. Biochemistry 45: 13970-13981.
2. Leonard TA, Rozycki B, Saidi LF, Hummer G, Hurley JH (2011) Crystal structure and allosteric activation of protein kinase C betaII. Cell 144: 55-66.
3. Marley J, Lu M, Bracken C (2001) A method for efficient isotopic labeling of recombinant proteins. J Biomol NMR 20: 71-75.
